# Interpreting Subjective and Objective Measures of Job Resources: The Importance of Sociodemographic Context

**DOI:** 10.3390/ijerph16173058

**Published:** 2019-08-23

**Authors:** Lauren L. Schmitz, Courtney L. McCluney, Amanda Sonnega, Margaret T. Hicken

**Affiliations:** 1 Robert M. La Follette School of Public Affairs, University of Wisconsin-Madison, WI 53706, USA; 2 Darden School of Business, University of Virginia, Charlottesville, VA 22903, USA; 3 Institute for Social Research, University of Michigan, Ann Arbor, MI 48106, USA

**Keywords:** healthy aging, work, occupational stress, occupational health, socioeconomic factors, data accuracy, demography

## Abstract

Salutary retirement policy depends on a clear understanding of factors in the workplace that contribute to work ability at older ages. Research in occupational health typically uses either self-reported or objective ratings of the work environment to assess workplace determinants of health and work ability. This study assessed whether individual characteristics and work-related demands were differentially associated with (1) self-reported ratings of job resources from older workers in the Health and Retirement Study, and (2) corresponding objective ratings of job resources from the Occupational Information Network (O*NET). Results from regression and relative weights analyses showed that self-reported ratings were associated with self-reported job demands and personal resources, whereas corresponding O*NET ratings were associated with differences in gender, race, or socioeconomic standing. As a result, subjective ratings may not capture important aspects of aging workers’ sociodemographic background that influence work ability, occupational sorting, opportunities for advancement, and ultimately the job resources available to them. Future studies should consider including both subjective and objective measures to capture individual and societal level processes that drive the relationship between work, health, and aging.

## 1. Introduction

The American workforce is aging, with 22.4% of full-time workers over 55 years of age in 2016, compared with 13.1% in 2000 [1]. Because the work environment is linked to aspects of quality of life such as job satisfaction [2] and mental and physical health [3,4], employers need to consider the ways in which workplaces can adapt to meet the needs of older workers. Workplaces that facilitate a happy and healthy older workforce may increase labor force retention, job engagement [5] and occupational health [6]. 

The literature suggests that there is a balance between job demands that require sustained physical or psychological effort and job resources that promote learning and engagement, and that greater job demands relative to job resources result in burnout [7]. Economic, social, psychosocial, and organizational resources available to employees may be a particularly important feature of the workplace, as these resources have been linked to more job satisfaction and engagement in the workplace [8]. Perhaps as important, however, is that the resources available to workers may offset the burden of job demands [9]. 

To date, the vast majority of research on the link between job demands, job resources, and worker well-being has relied on measures captured through self-reports. These subjective measures are useful for understanding worker agency and engagement. For example, although the physical and organizational aspects of one’s job may be beyond one’s control, social and psychological aspects are realized through individual perception and experience. If workers perceive that they have autonomy, skill variety, and opportunities for growth, they may have motivation to persist despite exposure to draining job demands [10]. 

However, objective measures of the workplace environment may also provide unique and useful information not captured by subjective self-reports. Objective data on workplace settings are available through the Occupation Information Network (O*NET). O*NET is a comprehensive database of job characteristics produced by the U.S. Department of Labor’s Employment and Training Administration and is the leading data source on job ratings [11]. O*NET ratings of workplace characteristics are assigned by occupational analysts and are based on information obtained from randomly surveying a broad range of workers within each occupational category. As such, these ratings could be considered a population average of job demands and resources that workers experience within a given occupation. In the context of an aging workforce, researchers are increasingly utilizing objective data on workplace settings to study later life well-being. For example, using O*NET information linked to surveys, previous research suggests that workplace environment is related to health disparities [12,13], later-life cognition [14], workplace injuries [15], and later-life employment transitions [16,17].

Nevertheless, neither subjective nor objective measures are without limitations. For example, self-reports may lead to inflated or biased associations between job demands and resources if the same worker is providing all of the information on the work environment (i.e., common method bias), or if they are not accurately perceiving their work environment due to unmeasured dispositional traits [18,19,20], affect [19,20], mental state [21], or other characteristics. Furthermore, racial/ethnic, gender, and socioeconomic occupational segregation means that women and non-White racial/ethnic and lower socioeconomic groups are more likely to occupy jobs with greater job demands and fewer resources than other groups, regardless of how these demands and resources are perceived, e.g., [22]. This segregation has important implications for effective interventions to reduce job strain and promote worker well-being. On the other hand, the downside to O*NET ratings is they do not capture the heterogeneous nature of workplace experiences within a given occupation, which can directly affect how an individual experiences work [23]. Thus, analyses of workplace characteristics that affect worker well-being and labor force attachment may benefit from research that includes subjective as well as objective measures [13,17]. 

To determine the distinction and utility of subjective and objective measures for future research, the purpose of this study was to evaluate whether subjectively and objectively rated job resources in older workers were differentially associated with a common set of personal resources, job demands, and sociodemographic characteristics. Given the increasing use of O*NET data on job characteristics, it is important to characterize these associations because factors that predict O*NET job resources may differ from factors that predict individual reports of job resources. Both self-reported job demands and personal resources such as health or personality attributes were hypothesized to be associated with self-reported job resources, while demographic characteristics were hypothesized to be more strongly associated with objective ratings of job resources. 

### 1.1. Description of the Job Demands–Resources (JD–R) Model: The Contribution of Subjective and Objective Measures

To guide the current study, the Job Demands–Resources (JD–R) model was used. JD–R has influenced decades of research in occupational health and safety and informed workplace health and safety programs in organizations [24,25]. The current JD–R model seeks to predict how worker motivation and strain affect job performance. According to JD–R, every job can be characterized by the presence of job demands and job resources [26]. Job demands refer to physical, psychological, social, or organizational aspects of the job that require sustained physical and/or psychological effort or skills leading to job strain [27]. Examples include high work pressure and emotionally demanding interactions with customers. Job demands are associated with physical health problems [28] and depression [29]. In contrast, job resources are physical, psychological, social, or organizational aspects of the job that stimulate personal growth, learning, and development leading to work engagement and motivation [26,30]. Examples of job resources include autonomy, skill variety, performance feedback, and opportunities for growth. 

Research indicates that an imbalance of high job demands relative to job resources results in exhaustion and burnout [31,32]. On the other hand, proportionately higher job resources are shown to buffer the negative association between job demands and burnout [32] and promote motivation to cope with stressful working conditions [9]. Subsequent versions of the JD–R model have been expanded to include personal resources (e.g., self-efficacy, self-esteem), which are presumed to increase engagement and mitigate the association between job demands and burnout [33,34]. 

Measures of job demands and resources overwhelmingly rely on self-reports. Capturing perceptions of the workplace are useful; individuals have a variety of experiences in the same job [35]. It is through these differences that scholars have identified mechanisms to create proactive changes to working conditions that foster gain (e.g., job crafting [36]) and mitigate loss (e.g., undermining [37]) spirals on the job [24]. However, studies that primarily use subjective measures may not be capturing the multi-level nature of organizations and their effect on worker outcomes. For example, job resources may be realized at the level of the organization at large (e.g., pay, career opportunities, job security), at the level of interpersonal or social relations (e.g., supervisor and co-worker support, team climate), by the organization of work (e.g., role clarity, participation in decision making), or at the level of the task (e.g., skill variety, task identity, task significance, autonomy, performance feedback) [7]. 

Moreover, subjective measures may not reflect societal level processes driven by sociodemographic characteristics such as gender, race, age, and socioeconomic status that are also “acting in the background” to influence the lived realities in workplaces. Evidence indicates sustained gender and racial occupational segregation are associated with multiple aspects of the employment process that result in lower quality jobs for women and non-White groups [22,38,39,40,41]. For example, jobs predominantly occupied by women and non-Whites not only have lower pay but also less flexibility, opportunities for advancement, and other resources compared to jobs predominantly occupied by White men [39,42,43,44]. Additionally, Black and Hispanic men and women are more likely to occupy jobs with hazardous exposures or fewer resources compared to their White counterparts [45]. Therefore, while subjective measures capture important aspects of the workplace, they leave gaps in our understanding of the ways in which social and organizational structures are related to the psychosocial reality of the workplace. 

Since O*NET ratings can be thought of as a population average of workplace characteristics for a given three-digit occupational code, they may be useful for capturing constructs across levels of analysis that also affect psychological phenomena unfolding within organizations. Thus, knowledge gathered from using objective data in addition to subjective data may help to guide the development of more population-based, effective interventions. To date, studies that have used objective indicators typically assess objective indicators of job demands, e.g., [46], since these are more easily assessed than objective measures of job resources. For instance, work hours, work overload, and time pressure are easily documented job demand metrics. In this study, we chose to compare four well-established subjective and objective measures of job resources (as opposed to job demands) because we were able to find nearly identical corollaries of these job resource measures in the HRS and O*NET.

### 1.2. Factors Predicting Subjective and Objective Measures of Job Resources 

Factors such as self-reported job demands, personal resources, and demographic characteristics that predict job resources are important to clarify as they may moderate or mediate the job demand–job resource imbalance. With respect to job demands, the vast majority of studies show a strong inverse relationship between subjectively reported job demands and job resources [24]. However, past research has not assessed the relationship between subjective job demands and objective job resources. 

Personal resources generally refer to internal mechanisms that help individuals function, appraise situations positively, and deal with stress [10]. Examples of personal resources include self-efficacy, optimism, and self-esteem [24]. Personal resources influence perceptions of job resources and demands, often leading to higher levels of work engagement. Personal resources may also include aspects of personality, including the Big Five personality traits [47]. For example, extraversion is associated with multiple dimensions of organizational commitment [48,49], job proficiency in occupations requiring social interactions (i.e., sales) [50,51], and job satisfaction due to experiencing positive emotions [52,53]. In contrast, neuroticism predisposes individuals to greater experiences of negative emotions and distress, which is in turn associated with lower job satisfaction [50,52]. Characteristics of conscientiousness, namely self-discipline and achievement, strongly predict job performance [50], continuance commitment [48], and job satisfaction [54]. Additionally, a recent study suggests that personality traits may moderate the effects of non-monetary job characteristics (i.e., physical demands, computer skill requirements, job flexibility, and workplace age discrimination) on retirement [16]. Finally, openness to experience is generally related to higher workplace creativity [55] and increased worker performance in the face of change.

In addition to dispositional resources, physical and mental health contributes to workplace performance and appraisal, e.g., [56]. Studies show that perceptions of one’s health are stronger predictors of change in health status compared to objective health measures [57,58]. Altering one’s perception of stress (subjective indicator) also fundamentally changes objective physiological processes [59]. 

Finally, given their potent role in shaping life experiences and opportunities, divergent patterns of association between sociodemographic factors and different sources of workplace reports may occur for several reasons. First, measures of social stratification affect selection into work environments and work experiences, e.g., [60,61,62]. For example, Black men and women are substantially less likely to hold managerial positions at any point in their life compared to White men [39], positions which may provide more job resources to offset job demands. Furthermore, socioeconomic status and gender both shape access to and progress in occupational career tracks [63]. A practical implication of occupational sorting based on sociodemographic characteristics is that people who are like each other will find themselves in similar jobs. To exemplify, given geographic segregation, women who are K-12 teachers are likely to have other women as colleagues who share similar demographic and educational backgrounds [64]. Thus, their subjective perceptions of the resources available to them are likely to be similar to their peers. 

## 2. Materials and Methods 

### 2.1. Data Source

Information on self-reported job resources, demands, personal resources, and sociodemographic characteristics were collected from the Health and Retirement Study (HRS)—a nationally representative study of Americans over the age of 50 and their spouses (regardless of age) that was launched in 1992 [65]. The HRS introduces a new cohort of participants every six years and interviews around 20,000 participants every two years through voluntary in-person (baseline) and telephone interviews (follow-up). Income, education, wealth, occupation, and employment information are collected alongside data on self-assessed well-being and health (For demographic and socioeconomic information, we used the RAND HRS data file (Version O, 2016). The RAND HRS data file is an easy to use longitudinal data set based on the HRS data. It was developed at RAND with funding from the National Institute on Aging and the Social Security Administration, Santa Monica.). HRS is funded by the National Institute on Aging (NIA U01AG0097) and is housed at the University of Michigan (UM) Institute for Social Research.

Since 2006, HRS has used a mixed-mode design in which half of the core sample is randomly assigned to a face-to-face core interview enhanced with physical and biological measures and a psychosocial questionnaire, and the other half is assigned to a telephone core-only interview. The Psychosocial and Lifestyle Questionnaire (PLQ), which includes personality assessment, is left behind at the end of the enhanced in-home interview for participants to mail back to the project offices. In 2008 and 2010, the PLQ included workplace characteristics in the subsample of respondents who reported working for pay in 2008 or 2010. All respondents have provided written consent, and the study protocol has been approved by the UM Institutional Review Board (IRB).

To compare self-reports of job resources from the HRS with more objective evaluations of job resources, data from the 2008 and 2010 O*NET were linked to the HRS using restricted three-digit U.S. Census occupation codes [12]. Since O*NET job characteristics were categorized by the Standard Occupational Classification (SOC) system, SOC codes were converted to three-digit 2000 Census Occupational Categories to construct a panel that could be merged with the HRS (SOC codes were converted to 2000 Census occupational codes using a coding system provided by the National Crosswalk Service Center. A consistent set of occupation codes for Census years 1980 and 2000 was developed by Meyer and Osborne [66].) To account for industry effects, restricted three-digit industry codes in the HRS were used to harmonize 2000 Census industry codes into eight broad categories.

### 2.2. Participants

In the HRS, 24,220 individuals responded in 2008 or 2010. Of these, 10,569 were working part time or full time for pay. Among working respondents, the sample was restricted to 7098 individuals between the ages of 50 and 70 who were not self-employed. Of these, 3369 respondents participated in the PLQ in 2008 or 2010, and 3,305 had a three-digit Census occupation code that could be merged with information from the O*NET. To avoid any further attrition, missing information on specific job demands or personal resources were set equal to zero and an additional dichotomous variable was included in regression analyses for each variable and set equal to one if the observation was missing. The final analytic sample included 3,305 respondents aged 50–70 who reported working full-time or part-time for pay when they completed the PLQ in 2008 or 2010. 

### 2.3. Measures

#### 2.3.1. Subjective and Objective Job Resources 

To maximize statistical power, composite indicators of subjective and objective job resources were constructed by taking the average across items from the HRS and O*NET (Table 1). All job resource or job demand inputs into the composite score were equally weighted and coded in the direction of the variable name so that a high score reflected a high value of the variable.

The items assessed whether a worker had opportunities for advancement, whether or not their work was recognized, the degree of workplace autonomy, and decision latitude. Questions in the HRS were measured on a four-point Likert scale (1= strongly disagree and 4 = strongly agree). The questions in the O*NET were asked on a five-point Likert scale where jobs were assigned a value based on the extent to which the attribute is important for job performance (1 = attribute is not important and 5 = attribute is extremely important). Items in the HRS were reverse coded to match the direction of the O*NET variable and O*NET measures were standardized to correspond to the four-point Likert scale used in the HRS. The composite indicators yielded reliable measures (O*NET job resources α = 0.85; HRS job resources α = 0.62).

#### 2.3.2. Job Demands 

Individual job demand items from the HRS PLQ and the Chronic Work Discrimination scale from the PLQ [67] were used to assess subjective job demands. 

*Subjective job demands.* Individual job demand items from the HRS PLQ were measured on a four-point Likert scale (1 = strongly disagree and 4 = strongly agree). These items included physical demands (“my job requires a lot of physical effort”), cognitive demands (“my job requires intense concentration or attention”), emotional demands (“I often feel bothered or upset at my work”), work-home conflict (“the demands of my job interfere with my personal life”), job insecurity (“my job security is poor”), time pressure (“I am under constant time pressure to do a heavy workload”), and work overload (“considering the things I have to do at work, I have to work very fast”). To assess whether their associations with subjective and objective job resource ratings varied by the nature of the job demand (i.e., physical, psychological, or social), subjective job demand items were assessed separately (i.e., a composite score was not created). 

*Work discrimination.* To assess chronic work discrimination, PLQ respondents indicated how often they experienced a behavior across six items during the last 12 months using a six-point Likert scale (1 = never to 6 = almost every day). These included “How often are you unfairly given the tasks at work that no one else wants to do”, “How often are you watched more closely than others”, “How often are you bothered by your supervisor or coworkers making slurs or jokes about women or racial or ethnic groups”, “How often do you feel that you have to work twice as hard as others at work”, “How often do you feel that you are ignored or not taken seriously by your boss”, and “How often have you been unfairly humiliated in front of others at work”. A composite indicator was created by averaging across equally weighted items (α = 0.81) [68].

#### 2.3.3. Personal Resources 

Measures of dispositional characteristics and perceived mental and physical health were used to assess personal resources. 

*Personality traits*. Thirty-one items from the PLQ that were derived from the Midlife in the United States (MIDUS) survey and the International Personality Item Pool (IPIP) were used to evaluate the ‘Big 5’ personality traits [69]. Participants indicated how well a list of traits describes them on a four-point Likert scale (1 = a lot to 4 = not at all). Items were reverse-coded (where necessary) and averaged to indicate dimensions of personality [68]. The final score for a personality dimension was set equal to missing if more than half of the list of traits within that dimension had missing values [68]. Personality trait measures included Neuroticism (α = 0.71), Extroversion (α = 0.75), Agreeableness (α = 0.79), Conscientiousness (α = 0.65), and Openness to Experience (α = 0.76). 

*Physical and mental health status.* Two indicators of self-reported physical health were used in analyses. Participants indicated their overall self-reported health status (SRHS) using a five-point scale. To reduce the number of covariates in the model, SRHS was coded as being equal to “1” if the respondent reported “excellent” or “very good” health and “0” if the respondent reported “good”, “fair”, or “poor” health (putting “good” in the same category as “excellent” and “very good” did not alter the results). Participants also rated how difficult it was for them to perform mobility tasks across five behaviors to indicate functional limitations [70] using a six-point measure (0 = none of the tasks are difficult to 5 = all five tasks are difficult). Examples of the tasks include “walking several blocks,” and “climbing one flight of stairs.”

Two indicators of mental health were evaluated. First, depressive symptoms were assessed using the Center for Epidemiological Studies-Depression (CES-D) scale [71,72,73]. Participants indicated how often they experienced each item during the past month using a five-point Likert scale (1 = All of the time to 5 = None of the time). Five ‘negative’ indicators (e.g., “you felt everything was an effort”) were summed and deducted from the sum of two positive indicators (e.g., “you felt happy”) to construct an overall score. Second, performance on HRS episodic memory tasks was used as an indicator of cognitive health [74,75]. Respondents were asked to repeat back a list of 10 common words read by the interviewer immediately after hearing them (*immediate recall*) and after approximately five minutes (delayed recall). Scores range from 0 to 20 and were calculated as the sum of the number of words recalled at the immediate recall phase and the number of words recalled at the delayed recall phase. 

#### 2.3.4. Demographic and Socioeconomic Characteristics 

Demographic measures included age, whether the respondent was female, and race/ethnicity. Measures of socioeconomic status included years of education, earnings in 2010 dollars, household income in 2010 dollars, household wealth (in $100,000s of 2010 dollars), and two-digit U.S. Census occupational classifications.

#### 2.3.5. Controls 

All models controlled for HRS birth cohorts and a dichotomous indicator for full-time versus part-time work status. Specifications with demographic and socioeconomic characteristics also controlled for two-digit U.S. Census industry classifications. 

### 2.4. Data Analysis

Linear regression models were used to evaluate the relationships between subjective or objective job resources and respondents’ job demands, personal resources, and sociodemographic characteristics. The empirical model was estimated as follows:

$$JR_{i}=\alpha +JD_{i}^{\prime }\delta +PR_{i}^{\prime }\theta +SD_{i}^{\prime }\gamma +X_{i}^{\prime }\beta +\varepsilon_{i}$$

where $JR_{i}$ is either the HRS self-reported job resources score or the O*NET job resources score for employee i, $JD_{i}$ is the vector of job demands, $PR_{i}$ is the vector of personal resources, $SD_{i}$ is the vector of demographic and socioeconomic characteristics, and $X_{i}$ is the vector of controls. The job resource indicators are standardized to have a mean of zero and a standard deviation of one for regression analysis. Results were generated using the statistical program Stata, version 15.

After running the subjective and objective job resource regression specifications, relative weights analysis (RWA) [76,77,78] was used to determine the relative contribution of job demands, personal resources, and sociodemographic variables towards the respective model R^2^. RWA excludes any variance that is redundant among predictors, and is valuable when there is an interest in determining the unique contribution of a set of highly correlated predictors. Relative weights were calculated using code developed by Tonidandel and LeBreton [78] in the statistical program R, version 3.3.2.

## 3. Results

### 3.1. Descriptive Statstics

Descriptive statistics are presented in Table 2. Average age was 58.9 (standard deviation (SD) = 5.33) and the majority of workers were White (76%) followed by Black (16%), and other races/ethnicities (8%). Workers had 13.58 years of education on average (SD = 2.73), and 63% worked in white-collar occupations (i.e., executive, professional, sales, or clerical occupations). In terms of workplace characteristics, HRS respondents seem to be fairly satisfied with their work environment; on a scale of 1 to 4, average self-reported ratings were slightly higher than average O*NET rankings (2.87 versus 2.66, respectively).

Table 3 reports correlations between individual HRS and O*NET job resource inputs and their respective composite indicators. In general, HRS inputs are more highly correlated with each other than inputs into the O*NET score. The correlation between the HRS and O*NET job resource composite scores is low (0.11), indicating they may be capturing different aspects of the data.

### 3.2. Association between Job Demands and Objective or Subjective Job Resources

Analyses were run in parallel and presented in separate tables for each outcome of interest, first subjective resources (Table 4) then objective resources (Table 5). In both tables, regression results are reported from five specifications that gradually added more variables to the analysis. Column (1) shows results from a specification that tested associations between job resources and job demands. Columns (2) and (3) add personal resources (personality traits and physical and mental health status, respectively). Column (4) adds demographic and socioeconomic indicators that are either ascribed (i.e., age, race, and gender) or achieved (i.e., education, income, and wealth), and Column (5) adds controls for two-digit Census occupation and industry categories. 

Job demands were related to both objective and subjective job resources. However, the particular job demands that were associated, as well as the direction and magnitude of their association, varied considerably between the two models. As expected in the subjective job resources model, self-reported job demands were on average inversely related to self-reported job resources, and the magnitude and significance of these associations persisted across all five specifications. Specifically, job insecurity (β = −0.26; *p*-value < 0.01), time pressure (β = −0.12; *p*-value < 0.01), emotional demands (β = −0.24; *p*-value < 0.01), work-life conflict (β = −0.12; *p*-value < 0.01), and work discrimination (β = −0.29; *p*-value < 0.01) were all inversely associated with subjective job resources (Table 4, Column 5). 

Conversely, in the objective job resources model, the significant self-reported job demands were either completely different (i.e., physical demands (β = −0.10; *p*-value < 0.01) and work overload (β = −0.05; *p*-value < 0.01)) and/or were positively associated with O*NET ratings (i.e., cognitive demands (β = 0.06; *p*-value < 0.01) and time pressure (β = 0.04; *p*-value < 0.05)) (Table 5, Column 5). Furthermore, the magnitude and significance of the coefficients in the objective job resources model appeared to be driven in large part by sociodemographic characteristics (Table 5, Columns 4–5). 

In particular, the associations between subjective job demands (i.e., physical demands, job insecurity, time pressure, work-life conflict, and work discrimination) and the objective job resource indicator were either substantially reduced in magnitude or became insignificant after controlling for race, gender, education, and occupation. This is in strong contrast to the subjective job resource model, where little to no effect of sociodemographic characteristics was evident. Results from the RWA indicated that self-reported job demands predicted 81.7% of the variation in the subjective job resource score compared to only 10.3% of the variation in the objective job resource score (Table 6).

### 3.3. Association between Personal Resources and Objective or Subjective Job Resources

Personal resources were more strongly related to subjective job resources than objective job resources. For example, extroversion was positively associated with subjective job resources (β = 0.14; *p*-value < 0.01) but not objective job resources (Table 4 and Table 5, Column 5). Interestingly, openness to new experiences was positively associated with subjective (β = 0.09; *p*-value < 0.05) and objective (β = 0.07; *p*-value < 0.05) job resources (Table 4 and Table 5, Column 5), and explained an almost identical proportion of the model R^2^ in both models (~1.4%) (Table 6). Unexpectedly, agreeableness was inversely associated with objective job resources (β = −0.08; *p*-value < 0.05) (Table 5, Column 5).

Consistent with our hypotheses, RWA revealed that personal resources explained 12.6% of the variation in self-reported job resources compared to only 4.7% of the variation in objective job resources (Table 6). In both job resource models, the majority of the variation from personal resources was explained by personality traits (i.e., physical and mental health status were not significantly associated with job resources after controlling for the Big 5 personality dimensions).

### 3.4. Association between Sociodemographic Factors and Objective or Subjective Job Resources

Demographic and socioeconomic factors were associated more strongly with objective ratings of job resources than subjective ratings of job resources. The direction of the relationship between sociodemographic variables and objective job resources aligns with occupational stratification in the labor market by race and gender. 

Specifically, being a member of an underrepresented social group was associated with working in jobs that O*NET rated as having fewer job resources, as indicated by the 0.14 standard deviation decrease in job resources for women (*p*-value < 0.01) and the 0.10 standard deviation decrease for Blacks (*p*-value < 0.01) (Table 5, Column 4). On the other hand, educational attainment increased access to job resources; each year of education was associated with a 0.09 standard deviation increase in expert-rated job resources (*p*-value < 0.01) (Table 5, Column 4). The associations between gender and years of education persisted after including fixed effects for occupation and industry (Table 5, Column 5), but associations between race, income, wealth, and job resources did not, perhaps because these associations were in large part driven by race-related occupational stratification and/or occupation-specific income and wealth gradients. In the subjective job resource model, being Black was the only sociodemographic characteristic that contributed to lower self-reports of job resources (β = −0.06; *p*-value < 0.05) (Table 5, Column 5). 

In general, compared to the occupational average, O*NET ratings of health-enhancing job resources were significantly higher for workers in certain white collar jobs (i.e., professional or sales) and significantly lower for workers in blue collar (i.e., operators, fabricators, laborers) or service jobs in the objective job resource model (Table 5, Column 5). Conversely, in the subjective job resource model, only individuals in clerical and administrative jobs reported having significantly lower job resources relative to the occupational average (β = −0.12; *p*-value < 0.01) (Table 4, Column 5). Finally, sociodemographic characteristics explained the largest proportion of the variation in the O*NET job resource model (83% including industry), and an almost negligible proportion of the variation in the self-reported job resource model (4.4%) (Table 6).

## 4. Discussion

This study provided empirical evidence that subjective and objective measures of job resources demonstrate different patterns of association with a common set of self-reported job demands, personal resources, and sociodemographic characteristics. Consistent with past empirical studies that have used the JD–R model, self-reported job demands were negatively related to self-reported job resources and explained a higher proportion of the model variance than any other domain we observed. Conversely, we found that self-reported job demands were not as highly associated with O*NET-rated job resources, explained a small proportion of the model variance, and in some cases displayed a positive pattern of association. These findings suggest that workers’ perception of their work environment differed significantly from O*NET ratings of their work environment. 

Given that our study is cross-sectional, these results may in part be driven by common method variance. However, if common method variance were entirely driving the results, the finding of strong associations between self-reported cognitive demands, physical demands, work-overload, and objective job resource ratings would be unlikely. Thus, certain perceived job demands appear to be linked to broader trends in job resources that hold at the population-wide level. For example, physical and cognitive demands may be characteristics of the work environment that are consistently experienced by all workers within a given three-digit occupational code. 

Similarly, differential associations between personality traits and subjective and objective job resources were also observed. Extroversion was positively associated with perceived job resources but was insignificant in the O*NET model. One explanation for this finding is that extroverted individuals may be more engaged in crafting their jobs in ways that may increase job resources and/or positive perceptions of them (e.g., asking for more feedback or help; [23,36]). Openness to experience was also positively associated with both subjective and objective job resources. This suggests that intellectual curiosity and preference for variety, for example, may not only enhance positive perceptions of job resources but also drive selection into better work environments. Although O*NET measures were designed to be independent of individual worker characteristics, these results indicate that ratings may be partly driven by selection into jobs that match individual characteristics of workers [64,79].

A significant contribution of this study is the examination of sociodemographic characteristics in the context of the JD–R model. These characteristics explained a small proportion of the observed variation in self-reported job resources, but explained the vast majority of the observed variation in O*NET ratings. Controlling for respondents’ sociodemographic background decreased the strength and magnitude of the associations between self-reported job demands and O*NET-rated job resources, but had no impact on associations between self-reported job demands and self-reported job resources. Together, these results suggest that while race, gender, and socioeconomic status appear to have affected the stratification or selection of HRS workers into certain occupations, and as a result their O*NET ratings, these same circumstances did not affect workers’ perceptions of their work environment. 

This may in part reflect the difficulty of objectively rating one’s own work experience relative to the experiences of workers in a different occupational class or setting. Given that exposure to specific job demands and resources are embedded within a larger socioeconomic hierarchy, an individual with low socioeconomic status may not view their workplace experiences as being objectively better or worse than an individual with higher social standing because they can only compare their own experiences relative to those in similar socioeconomic environments. This interpretation is in line with previous research that showed sociodemographic characteristics are associated with differential perceptions of the same occupation [7]. Regardless, given the widespread documentation of health disparities by race and socioeconomic status, these results suggest that the sociodemographic context may be an under-specified dimension of the occupational health domain that deserves further research. 

### 4.1. Limitations

Limitations of these analyses should be mentioned. Primarily, since the HRS does not currently have longitudinal self-reports of job demands and resources, the study could only be conducted in a cross-section of older workers. Thus, associations are not causal because unobservable individual heterogeneity may be spuriously correlated with job resources. For example, although physical and mental health were controlled for, it is possible that attrition bias due to poor health or the retirement decision, whereby only the healthiest workers survive or continue working, may have biased results. In addition, the HRS is limited to a sample of older workers. The absence of more detailed information on average job characteristics across different age groups could in part explain the lack of congruency between subjective and objective job resource measures. Thus, longitudinal studies that can assess contributors to deviations in subjective and objective reports over time and across age groups would strengthen our findings considerably.

### 4.2. Implications and Future Directions

With the aging of the workforce, it is more important than ever to build an accurate understanding of all of the forces at play in determining the health and labor market outcomes of older workers. These findings imply that subjective and objective ratings of the work environment are not interchangeable and may be capturing different aspects of individual and societal level processes that influence the relationship between work and health. As a result, choice of measure should be driven in part by whether the research question at hand is related to underlying differences in occupational characteristics that affect all workers or perceptual differences that may be more worker-specific. In addition, when possible, research should incorporate subjective and objective measures of the same workplace dimension, since choice of measure may impact findings on job strain, well-being, and worker health. For example, recent research using subjective and objective data on job characteristics from the HRS and O*NET found that even when items were matched as closely as possible across sources, they predicted retirement timing differentially [17]. 

Researchers may also want to use objective data sources to replicate findings with self-reported measures. For example, openness to new experiences was the only personality trait that was significant across both models, indicating that it may be a particularly robust predictor of the JD–R relationship and the psychological health of workers. Finally, perceptions of fairness, mistreatment, sexual harassment, and discrimination are currently understudied as subjective measures of job demands. Including measures of discrimination may not only deepen our understanding of individual workplace experiences, but may also indicate how current organizational structures create inequitable work environments. 

## 5. Conclusions

These findings stress the importance of including demographic and socioeconomic indicators within occupational health research. Evidence suggests that these worker characteristics are not just a source of variation that needs to be controlled for, but rather a resource that in itself may directly moderate or mediate the job demand–job resource imbalance. Previous research using self-reports of job demands or resources may not have captured the importance of the sociodemographic context because studies have largely been focused on assessing relationships between work and health at the individual level and may, therefore, have missed broader trends between groups. As a result, future work should examine the extent to which job demand–resource ratings are nested not just at the organizational level, e.g., [80], but also at the societal level to more accurately capture the complexity of the psychosocial workplace climate.

## Figures and Tables

**Table 1:** Description of HRS and O*NET job resources.

Job Resource	HRS Wording	O*NET Wording
Advancement	My job prospects are poor.	Workers on this job have opportunities for advancement.
Work recognized	I receive the recognition I deserve for my work.	Workers on this job receive recognition for the work they do.
Decision freedom	I have very little freedom to decide how I do my work.	How much decision-making freedom without supervision does the job offer?
Autonomy	At work, I feel I have control over what happens in most situations.	Workers on this job plan their work with very little supervision.

*Note*. HRS = Health and Retirement Study; O*NET = Occupational Information Network; Questions in the HRS are asked on a four-point Likert scale (1 = strongly disagree to 4 = strongly agree). O*NET assigns scores on a five-point Likert scale (1 = not important to job performance to 5 = extremely important to job performance). We rescaled the O*NET scores to match the four-point scale in the HRS. We reverse-coded “Advancement” and “Decision Freedom” in the HRS to match the O*NET variables.

**Table 2:** Descriptive statistics.

Variables	Mean	SD	Min	Max	N
Job resources composite score					
HRS	2.87	0.58	1	4	3305
O*NET	2.66	0.36	1.79	3.85	3305
Job demands					
Physical demands	2.20	1.11	1	4	3241
Cognitive demands	3.44	0.80	1	4	2406
Job insecurity	1.97	0.86	1	4	3210
Time pressure	2.14	0.94	1	4	3182
Emotional demands	1.92	0.76	1	4	3239
Work overload	2.52	0.82	1	4	3212
Work-life conflict	1.93	0.79	1	4	3144
Work discrimination	1.81	0.94	1	6	3296
Personal resources: Personality					
Neuroticism	2.03	0.60	1	4	3288
Extroversion	3.21	0.55	1	4	3291
Agreeableness	3.54	0.48	1	4	3292
Conscientiousness	3.47	0.42	1.6	4	3288
Openness to new experiences	3.00	0.52	1	4	3284
Personal resources: Physical/mental health					
Self-reported health status	0.15	0.35	0	1	3305
Total recall score	11.13	2.88	1	20	3237
CES-D score	1.03	1.63	0	8	3238
Mobility	0.52	0.96	0	5	3305
Demographic characteristics					
Age	58.99	5.33	50	70	3305
Works full time	0.75	0.43	0	1	3305
Female	0.59	0.49	0	1	3305
White	0.76	0.43	0	1	3305
Black	0.16	0.37	0	1	3305
Other race	0.08	0.26	0	1	3305
Socioeconomic status					
Years of education	13.58	2.73	0	17	3305
Individual earnings ($2010)	47,106	47,566	0	650,000	3305
Household income ($2010)	90,773	84,293	0	1,790,100	3305
Household wealth ($100,000s)	3.50	6.37	−8.61	114.96	3305
Occupation					
Executive, administrative, and managerial	0.13	0.34	0	1	3305
Professional, specialty, and technical	0.22	0.41	0	1	3305
Sales	0.08	0.27	0	1	3305
Clerical and administrative support	0.20	0.40	0	1	3305
Mechanical, construction, precision	0.07	0.25	0	1	3305
Operators, fabricators, and laborers	0.11	0.32	0	1	3305
Farming, forestry, and fishing	0.01	0.07	0	1	3305
Service	0.18	0.39	0	1	3305

*Note.* SD = standard deviation; HRS = Health and Retirement Study; O*NET = Occupational Information Network; CES-D = Center for Epidemiologic Studies-Depression Scale.

**Table 3:** Correlations between HRS and O*NET job resource variables and job resource composite indicators.

	Measure	1	2	3	4	5	6	7	8	9
1	Autonomy (HRS)									
2	Work recognized (HRS)	0.44 ***								
3	Decision freedom (HRS)	0.35 ***	0.31 ***							
4	Advancement (HRS)	0.21 ***	0.30 ***	0.19 ***						
5	Autonomy (O*NET)	0.12 ***	0.08 ***	0.12 ***	0.03 *					
6	Work recognized (O*NET)	0.11 ***	0.07 ***	0.10 ***	0.03 *	0.84 ***				
7	Decision freedom (O*NET)	0.10 ***	0.07 ***	0.08 ***	0.07 ***	0.64 ***	0.55 ***			
8	Advancement (O*NET)	0.03 *	0.03 *	0.04 **	−0.01	0.60 ***	0.68 ***	0.25 ***		
9	HRS JR score	0.70 ***	0.74 ***	0.67 ***	0.66 ***	0.12 ***	0.11 ***	0.11 ***	0.03	
10	O*NET JR score	0.11 ***	0.08 ***	0.10 ***	0.03 *	0.94 ***	0.93 ***	0.68 ***	0.77 ***	0.11 ***

*Note.* HRS = Health and Retirement Study; O*NET = Occupational Information Network; JR = job resources. Numbers in parentheses are internal consistency reliability estimates (Chronbach’s alpha). * *p* < 0.10, ** *p* < 0.05, *** *p* < 0.01.

**Table 4:** Regression analysis predicting subjective HRS job resources.

Variable	1	2	3	4	5
Job demands					
Physical demands	0.01 (0.01)	0.01 (0.01)	0.01 (0.01)	0.02 (0.01)	0.02 (0.02)
Cognitive demands	0.02 (0.02)	0.01 (0.02)	0.01 (0.02)	0.01 (0.02)	0.01 (0.02)
Job insecurity	−0.29 *** (0.02)	−0.27 *** (0.02)	−0.27 *** (0.02)	−0.26 *** (0.02)	−0.26 *** (0.02)
Time pressure	−0.11 *** (0.02)	−0.11 *** (0.02)	−0.11 *** (0.02)	−0.11 *** (0.02)	−0.12 *** (0.02)
Emotional demands	−0.27 *** (0.02)	−0.25 *** (0.02)	−0.25 *** (0.02)	−0.25 *** (0.02)	−0.24 *** (0.02)
Work overload	0.03 (0.02)	0.02 (0.02)	0.02 (0.02)	0.02 (0.02)	0.02 (0.02)
Work-life conflict	−0.11 *** (0.02)	−0.10 *** (0.02)	−0.10 *** (0.02)	−0.11 *** (0.02)	−0.12 *** (0.02)
Work discrimination	−0.30 *** (0.02)	−0.30 *** (0.02)	−0.30 *** (0.02)	−0.30 *** (0.02)	−0.29 *** (0.02)
Personal resources					
Neuroticism		−0.04 (0.03)	−0.04 (0.03)	−0.05 * (0.03)	−0.04 (0.03)
Extroversion		0.15 *** (0.03)	0.15 *** (0.03)	0.15 *** (0.03)	0.14 *** (0.03)
Agreeableness		−0.03 (0.04)	−0.03 (0.04)	−0.01 (0.04)	−0.00 (0.04)
Conscientiousness		0.03 (0.04)	0.03 (0.04)	0.02 (0.04)	0.03 (0.04)
Openness		0.11 *** (0.03)	0.11 *** (0.03)	0.10 *** (0.03)	0.09 ** (0.03)
CES-D score			0.00 (0.01)	0.01 (0.01)	0.01 (0.01)
Self-reported health status			−0.01 (0.04)	−0.00 (0.04)	−0.01 (0.04)
Total recall score			0.00 (0.00)	−0.00 (0.01)	0.00 (0.01)
Mobility			−0.01 (0.02)	−0.00 (0.02)	−0.00 (0.02)
Demographic/socioeconomic					
Age				0.00 (0.00)	0.00 (0.00)
Female				−0.05 * (0.03)	−0.05 (0.03)
Black				−0.07 ** (0.03)	−0.06 ** (0.03)
Other race				0.07 * (0.04)	0.06 * (0.04)
Years of education				0.01 (0.01)	0.00 (0.01)
Log earnings ($2010s)				−0.01 (0.01)	−0.01 (0.01)
Log household income ($2010s)				0.02 (0.02)	0.02 (0.02)
Household wealth ($100,000s)				0.00 (0.00)	0.00 (0.00)
Professional, specialty, technical					−0.02 (0.04)
Sales					0.03 (0.06)
Clerical and administrative					−0.12 *** (0.04)
Mech./construction/prod.					−0.06 (0.06)
Operators, fabricators, laborers					−0.08 (0.05)
Service					−0.03 (0.04)
Farming, forestry, fishing					0.13 (0.17)
N	3305	3305	3305	3305	3305
R^2^	0.39	0.41	0.41	0.41	0.42

*Note.* HRS = Health and Retirement Study; O*NET = Occupational Information Network; CES-D = Center for Epidemiologic Studies Depression Scale. Omitted category for race is “white”; omitted category for occupation is “executive, administrative, and managerial”. Both variables are effect coded so that coefficients represent group differences from the grand mean. All models include controls for birth cohort and full time work status. Variables with missing observations include additional dichotomous controls for missingness. Model 5 controls for two-digit census industry codes. Robust standard errors are in parentheses. * *p* < 0.10, ** *p* < 0.05, *** *p* < 0.01.

**Table 5:** Regression analysis predicting objective O*NET job resources.

Variable	1	2	3	4	5
Job demands					
Physical demands	−0.29 *** (0.01)	−0.27 *** (0.02)	−0.25 *** (0.02)	−0.19 *** (0.02)	−0.10 *** (0.01)
Cognitive demands	0.10 *** (0.02)	0.08 *** (0.02)	0.08 *** (0.02)	0.09 *** (0.02)	0.06 *** (0.02)
Job insecurity	−0.07 *** (0.02)	−0.06 *** (0.02)	−0.05 ** (0.02)	−0.02 (0.02)	−0.01 (0.02)
Time pressure	0.15 *** (0.02)	0.14 *** (0.02)	0.13 *** (0.02)	0.10 *** (0.02)	0.04 ** (0.02)
Emotional demands	−0.06 *** (0.02)	−0.04 * (0.02)	−0.03 (0.02)	−0.04 (0.02)	−0.01 (0.02)
Work overload	−0.05 ** (0.02)	−0.06 ** (0.02)	−0.06 ** (0.02)	−0.06 *** (0.02)	−0.05 *** (0.02)
Work-life conflict	0.10 *** (0.02)	0.09 *** (0.02)	0.09 *** (0.02)	0.04 * (0.02)	0.01 (0.02)
Work discrimination	−0.06 *** (0.02)	−0.06 *** (0.02)	−0.05 ** (0.02)	−0.02 (0.02)	−0.02 (0.02)
Personal resources					
Neuroticism		0.01 (0.03)	0.02 (0.03)	0.01 (0.03)	0.03 (0.02)
Extroversion		−0.10 *** (0.04)	−0.10 *** (0.04)	−0.05 (0.04)	−0.02 (0.04)
Agreeableness		−0.18 *** (0.04)	−0.17 *** (0.04)	−0.09 ** (0.04)	−0.08 ** (0.03)
Conscientiousness		0.14 *** (0.04)	0.11 ** (0.04)	0.07 * (0.04)	0.05 (0.03)
Openness to new experiences		0.31 *** (0.04)	0.29 *** (0.04)	0.16 *** (0.04)	0.07 ** (0.03)
CES-D score			−0.01 (0.01)	0.00 (0.01)	0.01 (0.01)
Self-reported health status			−0.07 (0.05)	0.03 (0.05)	0.03 (0.04)
Total recall score			0.03 *** (0.01)	0.01 (0.01)	0.01 (0.00)
Mobility			−0.04 ** (0.02)	−0.01 (0.02)	−0.01(0.01)
Demographic/socioeconomic					
Age				0.00 (0.00)	0.00 (0.00)
Female				−0.14 *** (0.04)	−0.10 *** (0.03)
Black				−0.10 *** (0.03)	−0.02 (0.03)
Other race				0.06 (0.04)	0.04 (0.03)
Years of education				0.09 *** (0.01)	0.04 *** (0.01)
Log earnings ($2010s)				0.02 ** (0.01)	0.01 (0.01)
Log household income ($2010s)				0.05 **(0.02)	0.01 (0.01)
Household wealth ($100,000s)				0.01 *** (0.00)	0.00 (0.00)
Professional, specialty, technical					0.36 *** (0.04)
Sales					0.66 *** (0.05)
Clerical and administrative					−0.31 *** (0.04)
Mech./construction/prod.					0.04 (0.06)
Operators, fabricators, laborers					−0.75 *** (0.04)
Service					−0.73 *** (0.04)
Farming, forestry, fishing					−0.02 (0.15)
N	3305	3305	3305	3305	3305
R^2^	0.17	0.20	0.21	0.28	0.52

* *p* < 0.10, ** *p* < 0.05, *** *p* < 0.01.

**Table 6:** Relative weights analysis for subjective HRS and objective O*NET job resource models.

Variable	Subjective Model (R^2^ = 0.42)		Objective Model (R^2^ = 0.52)	
	Raw Weight	% R^2^	Raw Weight	% R^2^
**Job demands**				
Physical demands	0.001	0.31	0.040 *	7.86
Cognitive demands	0.000	0.10	0.000	0.05
Job insecurity	0.078 *	19.33	0.001	0.11
Time pressure	0.033 *	8.29	0.006 *	1.20
Emotional demands	0.066 *	16.36	0.000	0.07
Work overload	0.011 *	2.63	0.001	0.13
Work-life conflict	0.028 *	7.02	0.003 *	0.57
Work discrimination	0.111 *	27.65	0.001 *	0.27
Total percent of model R^2^		81.69		10.26
**Personal resources**				
Neuroticism	0.013 *	3.34	0.000	0.06
Extroversion	0.013 *	3.20	0.001 *	0.26
Agreeableness	0.004 *	0.94	0.002 *	0.32
Conscientiousness	0.003 *	0.83	0.003 *	0.54
Openness to new experiences	0.006 *	1.46	0.007 *	1.42
Self-reported health status	0.006 *	1.42	0.002 *	0.32
Total recall score	0.003 *	0.78	0.002 *	0.39
CES-D score	0.000	0.07	0.005 *	1.04
Mobility	0.002	0.54	0.002 *	0.35
*Total percent of model R^2^*		12.59		4.68
**Demographic characteristics**				
Age	0.002	0.59	0.000 *	0.06
Female	0.001	0.19	0.005 *	0.94
Black	0.002	0.42	0.004 *	0.72
Other race	0.001	0.13	0.002 *	0.37
Total percent of model R^2^		1.33		2.10
**Socioeconomic status**				
Years of education	0.001	0.21	0.050 *	9.70
Individual earnings	0.001	0.13	0.009 *	1.84
Household income	0.001	0.37	0.015 *	2.87
Household wealth	0.002	0.40	0.007 *	1.34
Total percent of model R^2^		1.10		15.75
**Occupation**				
Professional, specialty, and technical	0.000	0.08	0.019 *	3.76
Sales	0.000	0.10	0.019 *	3.73
Clerical and administrative support	0.003 *	0.82	0.037 *	7.17
Mech./construction/precision prod.	0.001	0.18	0.019 *	3.62
Operators, fabricators, and laborers	0.002	0.39	0.080 *	15.49
Service	0.001	0.22	0.107 *	20.87
Farming, forestry, and fishing	0.001	0.25	0.039 *	7.62
*Total percent of model R^2^*		1.79		62.26

*Note.* See Table 5. Controls for full time status, industry (4.95%), and birth cohort account for the remainder of the model R^2^. * *p* < 0.05.
